# Practical Notes and Formulæ

**Published:** 1880-05-20

**Authors:** 


### PRACTICAL NOTES AND FORMULÆ.

**Hemorrhagic Malarial Fever.**—Dr. E. M. Parham, of Arkansas, writes:

I have found the following the best treatment for swamp or hemorrhagic fever, viz: Calomel and Dover's in small quantities, repeated every four hours, to correct morbid secretion and equalize the circulation, alternated with ten-drop doses of the fluid extract of ergot. Should the stomach be too irritable to bear the ergot, I use the tincture of the muriate of iron. I have found the quinine treatment totally inadmissible.

**Hay Fever.**—Dr. J. S. Stedham, of Georgia, writes:

I would like to hear from some of your contributors, through the Record, something on hay fever; its general prognosis, treatment, etc.

[We hope that the Doctor's request will meet with attention.—ED. RECORD.]

**Gonorrhœa—A New Treatment.**—

| | | | |
|---|---|---|---|
| ℞ | Brom. potass | ʒ ij. | |
| | Aquæ camph | ℥ iij. | |
| | Tinc. gelsem | ʒ ij. | M. |

Take a teaspoonful morning and noon, and two teaspoonsful at bed time, to quiet the nerves, soothe the mucous membrane of the urethra, and prevent chordee.

In conjunction with this treatment introduce gently three times a day a bougie of moderate size, four or five inches into the penis, well smeared with vaseline and bismuth.

| | |
|---|---|
| Subnit. bismuth | ʒ j. |
| Vaseline | ℥ j. |

Let this be done immediately after urinating, that the pus may be removed. Let the patient take a dose of salts before commencing this treatment; let him bathe the parts night and morning, and by the time the nervine mixture is consumed, and sometimes before, he will be well.

**For Irregular Menstruation.**—The following is a valuable remedy for scanty, irregular menstruation, when associated with and dependent upon anemia, neuralgia and neurasthenia:

| | | | |
|---|---|---|---|
| ℞ | Tinc. ferri chl | ʒ x. | |
| | Liquor potassa arsenitis | ʒ ij. | M. |

Dose, 12 drops after each meal, through a glass tube, in about one-third glass of water.—*Indep. Pract.*

**Cubebs in Whooping Cough.**—Dr. M. Sanchez has obtained rapid cures in cases of whooping-cough by the administration of an ethereal tincture of cubebs in doses of four or five drops three times a day.—*Gaceta Medica.*

**Compound Solution of Carbolic Acid.**—Hager recommends the following:

| | | |
|---|---|---|
| ℞ | Sumatra benzoin (2d quality) | 100.0 |
| | Aloes | 50.0 |
| | Crude salicylic acid | 25.0 |

Reduce to powder and add:

| | |
|---|---|
| Oil of spike | 50.0 |
| Oil of star anise | 10.0 |
| Alcohol | 1000.0 |

Macerate for one day, shaking occasionally, then add:

| | |
|---|---|
| Oleic acid | 100.0 |

and a previous prepared solution of

| | |
|---|---|
| Crude caustic soda | 60.0 |
| Borax | 25.0 |
| Water | 500.0 |

Digest, shaking occasionally, for one day, and add to the warm mixture:

| | |
|---|---|
| Crude carbolic acid (containing 90 to 95 per cent. phenol) | 3000.0 |

Shake for half hour, then set aside in a cold place for a week, and decant the liquid.

The solution must be used cautiously, so as not to come into contact with the eyes, lips and other tender portions of the body. For killing vermin on animals it is applied with a brush, previously diluted with 100 or 120 parts of water, and with linen or cotton, also for disinfecting offensive sores. When used for protecting horses, etc., against flies and other insects, very little of the composition is applied with a brush once or twice a day.—*Phar. Centralb.*

**Damiana.**—In painful hemorrhagic diseases of the kidneys and bladder, I have found nothing superior to:

| | | |
|---|---|---|
| ℞. | Damiana tinct | ℥ ij. |
| | Rhus. Glab. tinct | ℥ ij. |
| | Morphiæ Sulph | grs. iij. M. |

Sig.—Teaspoonful once in four hours.

In enlarged prostate, and acute and sub-acute inflammations of that gland, damiana in combination with phytolacca dec. and potassium bromide will be found very valuable:

| | | |
|---|---|---|
| ℞. | Damianæ tinct | ℥ ij. |
| | Phytolaccæ dec. tinct | ℥ i. |
| | Potassii Bromidi | ℥ ss. |
| | Syrupi Zingiberis | ℥ ss. |
| | Aqua dest. q. s. ad | ℥ vi. |

Sig.—Dessertspoonful every four hours until patient is fully relieved: afterward about once in six or eight hours until cured.—*Med. Tribune.*

**Senecio Aureus in Rheumatism.**—For removing the rheumatic diathesis, Dr. N. S. Davis extols the life-root plant, senecio aureus. In a typical case of chronic rheumatic carditis he prescribes :

| | | |
|---|---|---|
| ℞ | Acid carbolic (crystal) | 0.40 |
| | Glycerin (pure) | 16.00 |
| | Tinc. gelsemium | 16.00 |
| | Tinc. digitalis | 32.00 |
| | Fl. ext. senecio aureus | 96.00 |

Dose, 5 grams, or an ordinary teaspoonful, in a little water, just before each meal and at bed-time.

The steady use of this, with due attention to diet and exercise, and the avoidance of all use of alcoholic drinks and tobacco, will probably do as much to counteract the rheumatic diathesis, regulate the action of the heart, improve digestion, and thereby prolong the life and usefulness of the patient, as any course of treatment we could suggest.—*Med. and Surg. Reporter.*

**Excipient for Quinia Pills.**—One of the best excipients, and one which is very commonly used, is a mass made with tartaric acid, water and flour. The usual proportions are :

| | |
|---|---|
| Tartaric acid | 120 grains. |
| Fine wheat flour | 60 " |
| Water | 5 drops. |

This quantity is sufficient to make a pill mass with one troy ounce of sulphate of quinia. Care must be taken lest more water be added than is absolutely necessary, as an excess will produce too soft a mass, which would eventually flatten out.—*New Remedies.*

**In Menopause.**—The following prescription is specially serviceable in the various manifestations which often accompany the menopause :

| | | | |
|---|---|---|---|
| ℞ | Sodii bromidi | ʒ iv. | |
| | Tinc. Nucis vomica | ʒ ij. | |
| | Elixir calisaya | ℥ ij. | |
| | Syrupi pruni Virg | ℥ j. | |
| | Elixir simplicis | ʒ vj. | M. |

Dose, two drachms two, three or four times a day, as needed.—*Chicago Med. Gazette.*

**Antidote for Carbolic Acid.**—Husemann recommended several years ago saccharated lime (a solution of caustic lime in sugar-water) for neutralizing the poisonous effect of carbolic acid, while Sanftleben claims to have found an antidote in sulphuric acid, which, according to his statement, enters into a not poisonous combination with carbolic acid; he prescribes the following :

| | | |
|---|---|---|
| ℞ | Dilute sulphuric acid | 10.0 |
| | Muc. of gum arabic | 200.0 |
| | Simple syrup | 30.0 |

Mix and give a tablespoonful every hour.—*Pharm. Ztschr.*

**Compound Wine of Iron and Calisaya.**—One of our correspondents sends us the following formula, which, he says, is "far superior to any or all elixirs as a general tonic :

℞ Cinchona bark, Calisaya .......... ℥ iv.

Gentian root .......... ℥ iv.

Fresh hops .......... ℥ iv.

Dandelion root .......... ℥ iv.

Wild cherry bark .......... ℥ iv.

Dogwood bark (cornus Florida) .......... ℥ iv.

Orange peel .......... ʒ j.

Cinnamon .......... ʒ j.

Grind to a coarse powder, mix intimately, moisten with sherry wine and glycerin (equal parts), pack in a percolator, and pour enough best sherry wine on top to obtain one quart of percolate. Then add, previously dissolved in hot water :

Pyrophosphate of iron .......... ½ ℥.

Mix the whole together.

**Spirit of Nitrous Ether.**—The difference between a good article and a sorry one is well shown in the preparation of nitrous ether as prepared by Messrs. Merrell, Thorp & Lloyd, an advertisement of which appears in this Journal. The remedy as usually found in the shops has little or no strength, on which account many practitioners have abandoned its use; and yet, when pure, as prepared by the above House, it is very efficient as a diuretic and cooling diaphoretic, highly useful in fevers, in strangury and irritable bladder, etc.

The following formula is suggested for the relief of high febrile exascerbations, especially when the urine is red and scanty :

℞ Spirit of nitrous ether, pure .......... ʒ ij.

Water .......... ℥ iv.

Tinc. aconiti .......... gtts. v.

Dose, one teaspoonful every hour.

**Pruritus Ani.**—During last summer I had a case of this kind which baffled all my endeavors, until I used the following prescription:

℞. Camphoræ; Chloral Hyd., aa .......... ʒ ss.

Ung. Petrolei .......... ʒ vij. M.

Sig.—Ointment.

This gave immediate relief, and a few applications only were needed; the itching was permanently allayed. Repeated experiences with it since that time have so satisfied me of its efficiency, that I venture to suggest it to the readers of your valuable journal, in the hope that they also may find it a "friend in need."—DR. JOHN H. PACKARD, *in Medical and Surgical Reporter.*

**For After-Pains.**

℞. Tinct. aconite .......... gtts. v.
Tinct. macrotys .......... gtts. x.
Water .......... ℥ iv.

A teaspoonful as often as necessary (every one, two or three hours) to give relief.

